# Memorandum

**Published:** 1868-04-01

**Authors:** Jos. K. Barnes

**Affiliations:** Surgeon General, U. S. Army; War Department; Surgeon General’s Office


### *Memorandum*

For the information of persons desirous of entering the Medical Corps of the U. S. Army.

[EXTRACTS FROM LAWS OF THE UNITED STATES.]

ACT OF CONGRESS, *Approved* JUNE 30, 1834.

"SEC. 1. *Be it enacted, etc.,* That from and after the passing of this Act, no person shall receive the appointment of Assistant Surgeon in the Army of the United States, unless he shall have been examined and approved by an Army Medical Board, to consist of not less than three Surgeons or Assistant Surgeons, who shall be designated for that purpose by the Secretary of War; and no person shall receive the appointment of Surgeon in the army of the United States, unless he shall have served at least five years as an Assistant Surgeon, and unless, also, he shall have been examined by an Army Medical Board constituted as aforesaid."

ACT OF CONGRESS, *Approved* JULY 28, 1866.

"SEC. 17. *And be it further enacted*, That the Medical Department of the Army shall hereafter consist of one Surgeon General * * * * One Assistant Surgeon General * * * * One Chief Medical Purveyor and four Assistant Medical Purveyors * * * * Sixty Surgeons, with the rank, pay and emoluments of Majors of Cavalry. One hundred and fifty Assistant Surgeons, with the rank, pay and emoluments of First Lieutenants of Cavalry, for the first three years' service, and with the rank, pay and emoluments of Captains of Cavalry after three years' service." * * * *

All candidates for appointment in the Medical Corps, must apply to the Surgeon General, U. S. Army, for an invitation to appear before the Medical Examining Board. The application must be in the hand writing of the candidate, stating age and birthplace, and be accompanied by testimonials from Professors of the College in which he graduated, or from other physicians of good repute. If the candidate has been in the Medical service of the Army during the war, the fact should be stated, together with his former rank, and time and place of service, and testimonials as to qualifications and character from officers with whom he has served should also be forwarded.

Candidates must be graduates of some regular Medical College, proof of which must be submitted to the Board before examination, and must be between 21 and 30 years of age.

The morals, habits, and physical and mental qualifications of each candidate will be subjects for careful examination by the Board, and a favorable report will not be made in any case in which there is a reasonable doubt.

The following will be the general plan of examination:

1. A short essay, either autobiographical or upon some professional subject — to be indicated by the Board.

2. Physical examination. This will be rigid, and each candidate will be required to certify "*that he labors under no mental or physical infirmity, nor disability of any kind, which can in any way interfere with the most efficient discharge of his duties in any climate.*"

3. Examination as to general aptitude and education.

4. Written examination on anatomy, physiology, hygiene, surgery, and practice of medicine.

5. Oral examination on each of the above mentioned subjects, and also on obstetrics, general pathology, chemistry, toxicology, medical jurisprudence and materia medica.

6. Clinical examination, medical and surgical, at a hospital.
7. Performance of surgical operations on the cadaver.

The Board will deviate from this general plan whenever necessary, in such manner as they deem best to secure the interests of the service.

The Board will report the merits of the candidates in the several branches of the examination, and their relative merit in the whole, according to which, if vacancies occur within two years thereafter, *the approved* candidates will receive appointments and take rank in the Medical Corps.

An applicant failing at one examination, may be allowed a second after one year, but not a third.

No allowance will be made for the expenses of persons undergoing examination, as this is an indispensable prerequisite to appointment, but those who are approved and receive appointments will be entitled to transportation on their obeying their first order.

If the result of the examination of a candidate be satisfactory, he will be offered a contract for duty as Acting Assistant Surgeon until such time as he can be appointed or commissioned as Assistant Surgeon.

The pay and emoluments of Surgeons and Assistant Surgeons are shown by the following table:

| | Pay per month. | No. of rations per day. | Amount of rations per month. | SERVANTS. No. for which pay is allowed. | SERVANTS. Am't allowed for pay per month. | SERVANTS. Am't allowed for clothing per mo'th. | SERVANTS. Am't allowed for rations per month. | SERVANTS. Total amount allowed per month. | Aggregate amount receivable. | FORAGE FURNISHED FOR HORSES WHEN ACTUALLY KEPT. In time of war. | FORAGE FURNISHED FOR HORSES WHEN ACTUALLY KEPT. In time of peace |
|---|---|---|---|---|---|---|---|---|---|---|---|
| Assistant Surgeon, under three years' service - - | $53.33 | 4 | $36 | 1 | $16 | $6.50 | $9 | $31.50 | $120.83 | 2 | 2 |
| Assistant Surgeon, over three years' service - - - - | 70.00 | 4 | 36 | 1 | 16 | 6.50 | 9 | 31.50 | 137.50 | 3 | 2 |
| Assistant Surgeon, over ten years' service - - - - | 70.00 | 8 | 72 | 1 | 16 | 6.50 | 9 | 31.50 | 173.50 | 3 | 2 |
| Surgeon, under ten years' service - - - - - - | 80.00 | 4 | 36 | 2 | 32 | 13.00 | 18 | 63.00 | 179.00 | 4 | 2 |
| Surgeon, over ten years' service - - - - - - - | 80.00 | 8 | 72 | 2 | 32 | 13.00 | 18 | 63.00 | 215.00 | 4 | 2 |

In addition to the above, Surgeons and Assistant Surgeons are allowed an additional ration per day, after the termination of every five years service.

Quarters and fuel, or commutation therefor, are also furnished to medical officers.

The number of vacancies now existing in the Medical Corps of the Army is thirty-nine. [*Vide* advertising pages.]

JOS. K. BARNES,
*Surgeon General, U. S. Army.*

WAR DEPARTMENT,

SURGEON GENERAL'S OFFICE,

*January* 1, 1868.

